# Jaundice in a Returning Traveler—A Rare Manifestation of *Mycoplasma pneumoniae* Infection: Case Report

**DOI:** 10.5811/cpcem.47139

**Published:** 2025-08-26

**Authors:** Andrea Molin, Molly Crowe, Brionna Matt, Jessica R. Jackson

**Affiliations:** * Lewis Katz School of Medicine at Temple University, Department of Infectious Diseases, Philadelphia, Pennsylvania; † Lewis Katz School of Medicine at Temple University, Department of Emergency Medicine, Philadelphia, Pennsylvania

**Keywords:** *hemolytic anemia*, *cold agglutinin disease*, Mycoplasma pneumoniae

## Abstract

**Introduction:**

Cold agglutinin hemolytic anemia is a rare but serious complication of infections, including *Mycoplasma pneumoniae*. This case highlights the importance of considering infectious causes in patients with unexplained hemolysis.

**Case Report:**

A 62-year-old previously healthy male developed jaundice, dyspnea, and fatigue three weeks after returning from South America. Labs showed hemolysis with agglutination, a positive direct Coombs test, and elevated cold agglutinin titers. *M pneumoniae* was identified via polymerase chain reaction, confirming the diagnosis. He required uncrossmatched blood transfusion and was treated with doxycycline, with clinical improvement over four days.

**Conclusion:**

This case underscores the need for emergency physicians to recognize *M pneumoniae*-induced hemolysis during periods of increased incidence and seasonal activity. Early diagnosis, targeted testing, and awareness of macrolide resistance are critical for timely intervention and improved outcomes.

## INTRODUCTION

Cold agglutinin disease is a rare form of autoimmune hemolytic anemia that is often triggered by infections, with *Mycoplasma pneumoniae* being one cause. The immune system produces cold agglutinins, typically immunoglobulin M antibodies that bind to red blood cells at low temperatures, leading to complement-mediated hemolysis. This case highlights a classic presentation of *M pneumoniae* infection, complicated by cold agglutinin hemolytic anemia in a previously healthy individual. It underscores the importance of considering infectious triggers in patients presenting with hemolysis. Early recognition is essential for emergency physicians to initiate prompt treatment of *Mycoplasma* infection and associated complications.

## CASE REPORT

A 62-year-old man with no significant past medical history presented to the emergency department (ED) with yellowing of his skin and sclera. His symptoms also included progressively worsening fatigue, dyspnea, fevers, and a productive cough. He first noticed these symptoms three weeks prior, during his flight home from South America. The patient had recently completed a two-week trip to South America, visiting Buenos Aires, Argentina; Montevideo, Uruguay; Santiago, Chile; and Viña del Mar, Chile. He stayed in urban environments and upscale hotels, where he consumed most of his meals. He denied insect bites or significant animal exposures. He did not take malaria prophylaxis, as it was not recommended for his destinations. Since returning to Philadelphia, he had not traveled outside the city. His only recent medication was occasional use of an over-the-counter cough medicine containing dextromethorphan. He denied tobacco, alcohol, recreational drug use, or new sexual partners.

On arrival, his vital signs were significant for tachycardia with a heart rate in the 130s and tachypnea with a respiratory rate in the mid-20s. Initial laboratory findings revealed a total bilirubin of 2.8 milligrams per deciliter (mg/dL) (reference range: 0.2–1.1 mg/dL), alkaline phosphatase of 103 units per liter (U/L) (34–104 U/L), alanine aminotransferase of 30 U/L (0–44 U/L), aspartate aminotransferase of 31 U/L (0–34 U/L), creatinine of 1.00 mg/dL (0.80–1.30 mg/dL), lactate dehydrogenase of 696 U/L (140–271 U/L), and haptoglobin <10 mg/dL (44–215 mg/dL), suggesting hemolysis. A complete blood count (CBC) could not be processed initially due to hemolysis of the sample. Urinalysis showed moderate bilirubin and elevated urobilinogen. Computed tomography of the chest, abdomen, and pelvis demonstrated diffuse tree-in-bud opacities in the mid to lower lung fields and hepatic steatosis, without biliary ductal dilatation or gallbladder abnormalities.

Two subsequent CBC samples were repeatedly hemolyzed. A third sample, warmed prior to testing, yielded a hemoglobin of 5.5 grams (g)/dL (14–17.5 g/dL), white blood cell count of 24.5 × 10^3^ per cubic millimeter (K/mm^3^) (4.0–11.0 K/mm^3^), and platelets of 739 K/mm^3^ (150–450 K/mm^3^). A peripheral blood smear showed significant red blood cell clumping and agglutination, which markedly decreased when the sample was warmed (Image).

A direct Coombs test returned positive, and a cold agglutinin titer was elevated at 1:320 (Figure 1). The patient’s blood was unable to be crossmatched due to agglutination. He was ultimately transfused one unit of un-crossmatched blood.

Following these findings, a respiratory pathogen panel by polymerase chain reaction (PCR) tested positive for *Mycoplasma pneumoniae*, confirming the diagnosis of cold agglutinin hemolytic anemia precipitated by *M pneumoniae* infection. The patient’s laboratory results were notable for a negative Epstein-Barr virus (EBV) plasma deoxyribonucleic acid (DNA) PCR, ruling out active EBV viremia. Additionally, leptospira DNA PCR was negative, as were three consecutive blood parasite smears (Table 1).

Infectious disease and hematology services were consulted by the ED and followed the patient during his inpatient stay. He was treated with supportive blood transfusion and a 14-day course of doxycycline 100 mg every 12 hours. His hepatic function tests and blood counts gradually improved during his four-day hospital stay (Table 2). He was discharged with instructions for close follow-up with his primary care physician.

*CPC-EM Capsule*

What do we already know about this clinical entity?

Mycoplasma pneumoniae *can trigger cold agglutinin-mediated hemolytic anemia, which may progress to life-threatening complications and requires early intervention*.

What makes this presentation of disease reportable?

*This case highlights the diagnostic hurdles and empiric management of* M pneumonia*-associated hemolytic anemia from an emergency medicine perspective*.

What is the major learning point?

*In patients with respiratory symptoms and hemolysis, consider* M pneumoniae*. Polymerase chain reaction for the bacteria and cold agglutinin testing enable prompt diagnosis and treatment*.

How might this improve emergency medicine practice?

*Underscores importance of broad hemolysis workup; if* M pneumoniae *is confirmed, begin early atypical antimicrobials and use warmed blood products if needed*.

## DISCUSSION

While cold antibody autoimmune hemolytic anemia secondary to *M pneumoniae* is a well-documented phenomenon in infectious diseases, this case offers unique insights from an emergency medicine perspective, particularly with the broad differential diagnosis that was considered and the acuity of the hemolytic anemia.

The potential causes of hemolytic anemia in an adult with a history of recent travel are numerous. While non-infectious causes such as congenital or inherited disorders, malignancies, systemic autoimmune diseases, and drug-induced conditions should be considered, the presence of hemolytic anemia in a returning traveler is particularly concerning for acute infections. In this context, vector-borne illnesses such as malaria, Carrion disease, babesiosis, and leptospirosis, as well as infection-associated hemolytic uremic syndrome, should be prioritized in the differential diagnosis.^1^ However, infectious causes of hemolytic anemia in the setting of cold agglutination most commonly involve *M pneumoniae* or Epstein-Barr virus. Although rare, *M pneumoniae* infections can progress to life-threatening extrapulmonary complications, in addition to severe hemolytic anemia including neurologic, mucocutaneous, cardiac and vascular complications, and renal failure.^2^–^4^

Furthermore, this case highlights the critical role of emergency physicians in evaluating and managing *M pneumoniae*-associated hemolytic anemia. Difficulty in obtaining accurate lab results due to hemolysis and agglutination may hinder diagnosis and treatment, particularly if transfusion is needed. Early initiation of targeted laboratory testing such as direct Coombs test and cold agglutinin titers facilitates the prompt identification of hemolytic anemia secondary to *M pneumoniae* infection. Empiric antibiotic treatment for *M pneumoniae* typically involves a macrolide or tetracycline. Interestingly, macrolide resistance in *M pneumoniae* has been gradually increasing since the early 2000s. The prevalence of resistance varies by region, with a global rate of 28% and a lower rate of 10% in the United States.^5^

If required, it is imperative to recognize that transfused blood should be warmed to 37° Celsius, and the number of transfusions should be minimized to prevent exacerbating ongoing hemolysis. Additional early interventions to consider may include folate supplementation and maintaining warmth in the extremities, nose, and ears to reduce cold-induced hemolysis.^6^

In 2023, *M pneumoniae* infections began to resurge globally after a period of relative inactivity during the COVID-19 pandemic. By early spring and late summer 2024, the number of cases surged, leading to a significant rise in ED visits with diagnoses of *M pneumoniae*. Although most cases were among children, adults were also notably affected. Data quantifying the percentage of individuals impacted by severe hemolytic anemia remain limited.^5^

## CONCLUSION

This case illustrates the complexities of diagnosing and managing *M pneumoniae*-associated hemolytic anemia in the emergency department, emphasizing the importance of a broad differential diagnosis, particularly in returning travelers. Early recognition and targeted lab testing are crucial for timely intervention and improved patient outcomes. With increasing macrolide resistance and a rising incidence of *M pneumoniae* infections, clinicians must remain vigilant for its potential life-threatening complications. Prompt consultation with hematology and infectious disease specialists can help mitigate morbidity and optimize management in complex cases.

## Figures and Tables

**Image:**
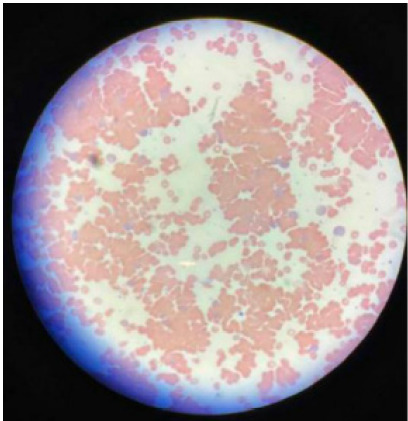
Room-temperature peripheral blood smear demonstrating near complete agglutination of red blood cells. Image credit to Temple University Hospital Department of Hematology.

**Table 1:** Specialty laboratory testing in patient who tested positive for *Mycoplasma pneumoniae*.

Laboratory Test	Result
Blood culture	No growth at five days
Cold Agglutinin Titer	1:320
EBV DNA Quantitative PCR (IU/mL)	Target not detected
EBV IgG	Positive
EBV IgM	Negative
Fourth Generation HIV Screen	Negative
Hepatitis C antibody	Positive
Hepatitis C RNA PCR (IU/mL)	Target not detected
Leptospirosis DNA PCR Qualitative	Target not detected
Mononucleosis screen	Negative
Blood parasite smear (hospital day 0)	No blood parasites seen
Blood parasite smear (hospital day 1)	No blood parasites seen
Blood parasite smear (hospital day 2)	No blood parasites seen
Respiratory pathogen panel by PCR	Positive for *Mycoplasma pneumoniae*; negative for all other organisms tested

*DNA* indicates deoxyribonucleic acid; *EBV*, Epstein Barr virus; *IgG*, immunoglobulin G; *IgM*, immunoglobulin M; *IU/mL*, international unit per milliliter; *PCR*, polymerase chain reaction; *RNA*, ribonucleic acid.

**Table 2:** Routine lab testing trends in patient hospitalized with *M pneumoniae*-induced hemolysis.

Laboratory Test	Hospital Day 0	Hospital Day 1	Hospital Day 2	Hospital Day 3	Hospital Day 4
**WBC (K/mm** **^3^** **)**	Hemolyzed sample	24.5	19.96	15.97	13.07
**HgB (g/dL)**	Hemolyzed Sample	5.5	7.3	7.3	7.7
**Plt (K/mm** **^3^** **)**	Hemolyzed Sample	739	743	647	619
**Cr (mg/dL)**	0.86	Not tested	0.97	0.82	0.94
**Tb (mg/dL)**	2.8	Not tested	3.1	1.8	1.4
**Db (mg/dL)**	Not tested	Not tested	0.7	0.4	0.3
**ALP (U/L)**	102	Not tested	30	25	36
**AST (U/L)**	28	Not tested	29	20	29
**ALT (U/L)**	28	Not tested	106	100	98
**Haptoglobin(mg/dL)**	Not tested	<30	<30	<30	<30
**LDH (U/L)**	Not tested	696	570	480	443

Abbreviations: *ALP*, alkaline phosphatase; *ALT*, alanine aminotransferase; *AST*, aspartate aminotransferase; *Cr*, creatinine; *Db*, direct bilirubin; *g*, gram; *HgB*, hemoglobin; *LDH*, lactate dehydrogenase; *mg/dL*, milligrams per deciliter; *Plt*, platelets; *K/mm^3^*, thousands per cubic millimeter; *Tb*, total bilirubin; *U/L*, units per liter; *WBC*, white blood count.
